# Is There Sufficient Local Evidence to Inform Biofortification Policies Against Micronutrient Deficiencies? A Global Concern for Food Security and Human Health

**DOI:** 10.3390/ijerph23020261

**Published:** 2026-02-19

**Authors:** Johan Camilo Vergara-Rios, Ivan David Lozada-Martinez, Juan David Reyes-Duque, Maria Trinidad Plaza Gómez

**Affiliations:** 1 Departamento de Ingeniería Agrícola y Alimentos, Universidad Nacional, Medellín 050034, Colombia; jcvergarar@unal.edu.co; 2 Biomedical Scientometrics and Evidence-Based Research Unit, Department of Health Sciences, Universidad de la Costa, Barranquilla 080002, Colombia; 3 Clínica Iberoamérica, Barranquilla 080002, Colombia; 4 Facultad de Ciencias para la Salud, Universidad de Manizales, Manizales 170001, Colombia; jreyes@umanizales.edu.co; 5 Facultad de Ingeniería, Ingeniería Industrial, Universidad de Córdoba, Montería 230002, Colombia

**Keywords:** malnutrition, deficiency diseases, biofortification, evidence gaps, developing countries, sustainable development

## Abstract

Micronutrient deficiencies remain a persistent challenge to global health and food security, particularly in low- and middle-income countries where evidence-based strategies are urgently needed. Biofortification of staple crops has been promoted as a complementary intervention to supplementation and food fortification, but its effective implementation requires locally relevant studies. Such evidence is essential because the performance and adoption of biofortified crops depend on context-specific factors, including crop varieties, soil micronutrient dynamics, dietary patterns, cultural acceptability, and bioavailability, which limit the transferability of findings across settings. This perspective examines whether countries with the highest micronutrient burdens generate sufficient local research to inform biofortification policy decisions. We conducted a bibliometric mapping of peer-reviewed literature indexed in Scopus and compared country-level publication counts with indicators of iodized salt coverage, zinc deficiency, and childhood anemia, which were selected because they are prioritized metrics in global health and food security. From 776 eligible articles, most publications originated from a small group of high- and middle-income countries, whereas regions facing the greatest nutritional burdens, including parts of Sub-Saharan Africa and South Asia, contributed little to the scientific output. Countries with low iodized-salt coverage, high zinc deficiency, or childhood anemia above 40% frequently showed zero or minimal publications. This misalignment suggests that countries facing the greatest nutritional vulnerabilities may be underrepresented in the indexed scientific literature. These findings highlight the value of further strengthening research participation and visibility in high-burden settings to ensure that the evidence base more accurately reflects global needs.

## 1. Introduction

Micronutrient deficiencies remain a challenge on global health and food security. They shape pregnancy outcomes, child growth and learning, and they intersect with food prices, regulation, and the reach of basic services [1]. Policies promise “evidence-based” action but that promise only holds if countries can point to a modest, local body of studies when they plan and budget [1,2,3].

Biofortification is the process of increasing the micronutrient content of staple crops through conventional plant breeding, agronomic practices, or modern biotechnological approaches, with the goal of improving the nutritional quality of foods at the point of production [4,5,6]. Biofortification has become an important strategy to address micronutrient deficiencies by increasing the micronutrient content of staple crops consumed daily by millions [2,6]. It is recognized as a practical complement to food fortification and supplementation, reaching rural and low-income populations that are often missed by other interventions [3]. As part of the global agenda on food security and human health, biofortification contributes directly to the Sustainable Development Goals, linking agriculture, nutrition, and health [7]. Continued research and development in this area is essential to strengthen adoption, improve nutritional sustainability, and accelerate progress against hunger and malnutrition worldwide [7].

It is important to distinguish between different types of evidence relevant to biofortification. Previously, research already demonstrates the biological and nutritional efficacy of biofortified crops across diverse settings, and such efficacy data do not need to be reproduced by every country [1,2,3]. What many countries often lack is locally generated, context-specific evidence needed to adapt and implement biofortification programs, including agronomic performance under local soil conditions, cultural acceptability, dietary patterns, supply-chain feasibility, and policy development [3]. Consequently, it is necessary to examine the availability of locally generated evidence rather than research production capacity or policymakers’ access to global evidence. Clarifying these distinctions is essential to avoid conflating global efficacy with the contextual information necessary for national decision-making [2,3].

Randomized effectiveness trials have demonstrated that vitamin A-rich, orange-fleshed sweet potato and other biofortified staples can improve vitamin A intakes and status among children and women in real-world settings in sub-Saharan Africa [4]. Likewise, cluster-randomized trials of zinc-biofortified wheat in rural Pakistan have shown higher zinc intakes and reductions in iron deficiency among adolescent girls consuming biofortified flour [5]. These findings are consistent with broader evidence syntheses from a systematic review, which conclude that biofortified crops can contribute to reducing vitamin A, iron, and zinc deficiencies in high-burden population [6].

The availability of scientific evidence on biofortification and nutritional deficiencies, and its relationship with global indicators of food security and global health had not been systematically quantified. Addressing this gap is essential to highlight where knowledge is lacking, to justify the design and implementation of future studies grounded in context-specific health and nutrition needs, and to guide a more objective allocation of resources in alignment with the global agenda on food security and human health [8].

Quantifying publication output at the country level is not intended to measure research quality or institutional capacity, but it provides a transparent proxy for the territorial availability of peer-reviewed evidence that can inform policy discussions [7,8]. Bibliometric indicators have been widely used to identify geographic inequities in scientific production because publication volume reflects, at minimum, whether a knowledge domain is being locally studied, documented, and disseminated [8]. While this approach does not capture deeper components of knowledge structures such as research infrastructure, funding systems, or policymaker engagement, it allows for systematic comparison across countries using standardized data [8]. Importantly, in fields such as nutrition and human health, even a modest body of locally generated publications can play a catalytic role in shaping policy adoption, guiding feasibility assessments, and attracting programmatic investment. For this reason, mapping where evidence exists, and where it is absent, provides practical insights for implementation planning and highlights contexts where decision-makers may lack accessible, country-relevant studies [8].

Building on this premise, this correspondence examines whether countries experiencing the greatest micronutrient burdens also show limited availability of locally linked, peer-reviewed research on biofortification and micronutrient deficiencies, and how this distribution of evidence aligns with some recognized global indicators of nutritional need. By comparing country-level publication output with indicators of iodine, zinc, and childhood anemia, the analysis highlights geographic mismatches between where evidence is produced and where it may be most relevant for informing discussions on implementation.

In this analysis, these indicators were not selected to imply a direct association between each indicator and biofortification itself. Rather, they serve as high-quality, globally standardized proxies of micronutrient vulnerability, nutritional inequity, and food security conditions, domains in which biofortification functions as one of several potential mitigation strategies.

## 2. Materials and Methods

A systematic search was conducted in Scopus, selected because it is the database that hosts the largest repository of peer-reviewed scientific literature. The search strategy was built using MeSH terms and their synonyms: (1) Biofortification (MeSH Unique ID: D000072759); (2) Malnutrition (MeSH Unique ID: D044342); and (3) Deficiency Diseases (MeSH Unique ID: D003677). The full search strategy was: (TITLE-ABS(Biofortification) OR TITLE-ABS(“Biofortified Crop*”)) AND (TITLE-ABS(Malnourishmen*) OR TITLE-ABS(Undernutrition) OR TITLE-ABS(“Nutritional Deficienc*”) OR TITLE-ABS(Malnutrition) OR TITLE-ABS(“Deficiency Disease*”) OR TITLE-ABS(Avitaminosis) OR TITLE-ABS(Avitaminoses) OR TITLE-ABS(“Vitamin Deficienc*”) OR TITLE-ABS(“Ascorbic Acid Deficienc*”) OR TITLE-ABS(Scurvy) OR TITLE-ABS(“Vitamin A Deficienc*”) OR TITLE-ABS(“Vitamin B Deficienc*”) OR TITLE-ABS(“Choline Deficienc*”) OR TITLE-ABS(“Folic Acid Deficienc*”) OR TITLE-ABS(Hyperhomocysteinemia) OR TITLE-ABS(Pellagra) OR TITLE-ABS(“Riboflavin Deficienc*”) OR TITLE-ABS(“Thiamine Deficienc*”) OR TITLE-ABS(“Vitamin B12 Deficienc*”) OR TITLE-ABS(“Vitamin B6 Deficienc*”) OR TITLE-ABS(“Vitamin D Deficienc*”) OR TITLE-ABS(“Vitamin E Deficienc*”) OR TITLE-ABS(“Vitamin K Deficienc*”) OR TITLE-ABS(“Magnesium Deficienc*”) OR TITLE-ABS(“Potassium Deficienc*”) OR TITLE-ABS(“Protein Deficienc*”) OR TITLE-ABS(“Protein-Energy Malnutrition”)).

The search was conducted on 18 September 2025. Only peer-reviewed publications published in regular journal issues were included, while book chapters, conference papers, books, retracted items, editorials, book series, trade journals, and in press articles were excluded. This ensured rigor in identifying the body of potentially actionable evidence available to inform decision-making. This methodology has previously been used to explore this same type of knowledge gap [9,10].

The final dataset was exported and standardized using Bibliometrix, v. 5.2.1. From this corpus, a country-level publication count was compiled, assigning one count to a country each time it appeared in a paper. No co-authorship networks or weighting methods were applied, keeping the analysis transparent and easy to interpret [9,10]. These publication counts were then compared, country by country, with the most recent national values for three indicators of nutritional deficiencies: household use of iodized salt (%) [11], prevalence of zinc deficiency [12], and share of children who have anemia [13]. All indicators were extracted from open-access datasets available in Our World in Data [11,12,13], specifically within their food security and nutrition categories. To ensure clarity, results were reported as frequencies and percentages, using straightforward thresholds: iodized-salt coverage < 70% (off-track) and <50% (severely off-track); zinc deficiency > 25% and >35%.

The thresholds used in this study were not defined by the authors. Each indicator is reported by its respective global data source using pre-established cut-points [11,12,13]. Because these categories originate from normative global frameworks, our analysis reproduced them exactly as reported in the source databases, and no sensitivity analysis was applied.

These indicators were selected because they are prioritized metrics in global health and food security monitoring, providing a standardized foundation for cross-country comparison [11,12,13]. Although iodized salt coverage reflects a fortification strategy rather than biofortification, it remains a foundational marker of micronutrient program implementation capacity [11]. Zinc deficiency directly relates to biofortifiable crops such as zinc-enriched wheat and rice [12]. Childhood anemia, while multifactorial, is widely used as a population-level indicator of micronutrient deprivation, dietary inadequacy, and nutritional vulnerability [13]. Together, these metrics offer a practical proxy for assessing the alignment between nutritional burden and the territorial availability of research on biofortification [11,12,13].

Country assignment was based on author affiliation as indexed in Scopus. Each country appearing at least once in a paper’s affiliation metadata received one count, following a whole-counting approach commonly used in territorial analyses of research availability. Thus, if a publication listed co-authors from five countries, each country received one count. This method quantifies the presence of a country in the peer-reviewed knowledge base, rather than the geographic location where the empirical work was conducted. In cases where a study was performed in a different country than the authors’ affiliations (such as a trial conducted in Kenya by researchers affiliated exclusively with Japanese institutions), the publication would be counted for Japan only, reflecting the absence of local institutional participation in the research process.

This decision was made because distinguishing research location from research ownership or local participation is not possible through bibliographic metadata alone, and attributing evidence to a country without local authorship risks overstating domestic research capacity. Fractional counting was not used because the objective of this analysis was not to measure contribution intensity but to identify whether a minimum body of locally linked evidence exists within countries experiencing high nutritional burdens. The limitations of this approach are acknowledged, but for the purposes of mapping territorial evidence gaps, whole counting provides a transparent and policy-relevant approximation of where scientific activity is institutionally situated.

We acknowledge that this approach may underrepresent evidence conducted in high-burden countries by international teams without local authorship, but this limitation is intrinsic to the available data and reinforces the value of evaluating territorial research participation.

Country-level authorship data were enriched using the World Bank’s official classification of economies [14]. Each country identified in the bibliometric dataset was matched with its corresponding geographic region [14] and income group [14] as defined by the World Bank. This categorization allowed us to aggregate publication counts and authorship frequencies both by region and by income group, enabling comparative analyses of research distribution across different socioeconomic and geographic contexts.

## 3. Unequal Distribution of Research and Nutritional Burden

**Figure 1:**
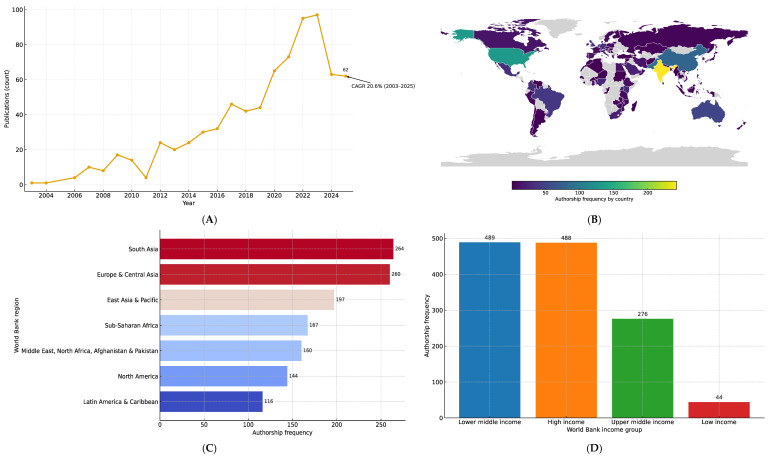
(**A**) Annual growth of publications (2003–2025) (N = 776). The curve highlights the long-term upward trend, with the compound annual growth rate (CAGR) of 20.6% indicating a sustained increase in scientific output over the study period. (**B**) Global authorship frequency by country in research on biofortification and micronutrient deficiencies. The map illustrates the distribution of international authorship across countries, represented as a heatmap. Darker shades indicate higher frequencies of co-authorship, while lighter shades indicate fewer collaborations. Gray areas correspond to countries without identified publications in the dataset. The internal color scale denotes authorship frequency. (**C**) Authorship frequency by World Bank regions. (**D**) Authorship frequency by World Bank income groups.

The initial Scopus search identified 1085 documents, but after applying the inclusion and exclusion criteria, a total of 776 papers remained for analysis. The temporal analysis revealed a consistent increase in the number of publications on biofortification and micronutrient deficiencies between 2003 and 2025, with an overall compound annual growth rate of 20.6% (Figure 1A). These papers were attributed to 91 countries, generating a corpus of 1308 country-linked authorships (Figure 1B).

When stratified by geography, the majority of publications were concentrated in South Asia, whereas Latin America, Sub-Saharan Africa and the Middle East contributed comparatively fewer collaborations (Figure 1C). In terms of income classification, high- and upper-middle-income countries together accounted for the largest share of publications, while low-income countries remained underrepresented. However, low- and middle-income countries, considered as a single group, exhibited the highest overall frequency when compared with each income category individually (n = 489) (Figure 1D).

The distribution of publications was highly concentrated: the median country contributed only 4 papers (interquartile range 2–14). A small number of countries dominated the field, with India (242 publications), the United States (130), Pakistan (89), China (79), and Australia (50) together accounting for 45.1% of all publications, while the top ten countries represented 58.7% (Supplementary Material). In practical terms, most countries have access to only a minimal body of context-relevant evidence.

When publication counts are compared with national iodized-salt coverage, the knowledge gap becomes evident. Using the most recent values available, 11 countries remain below 70% coverage (Supplementary Material). Within this group, the median publication count is 0, with 81.8% at or below the global median (≤4 papers) and the same proportion falling in the bottom quartile (≤2 papers). The situation is even more critical in the four countries below 50% coverage, where the median is 0 and 100% of them are both at or below the median and within the bottom quartile. Concrete examples include Gambia (17% coverage; 0 papers), Guinea-Bissau (38%; 0), Djibouti (44%; 0), North Korea (48%; 0), Moldova (58%; 0), Guatemala (59%; 0), South Sudan (60%; 0), Chad (65%; 0), and Somalia (69%; 0). These are precisely the contexts where straightforward public health measures, such as enforcing iodization standards, conducting retail-level testing, and improving market surveillance, would greatly benefit from basic country-level studies, yet such evidence remains largely absent.

Based on the most recent values, 34 countries report zinc deficiency levels above 25% (Supplementary Material). In this group, the median publication count is 0, with 79.4% at or below the global median (≤4 papers) and 70.6% in the bottom quartile (≤2 papers). The situation is particularly concerning in the nine countries exceeding 35% deficiency, where the median publication count is only 1, and the majority remain at or below the median (77.8%) and in the bottom quartile (55.6%). Notable examples include the Democratic Republic of Congo (54.3% deficiency; 0 papers), Lesotho (38.3%; 0), Burundi (38.0%; 0), Liberia (35.2%; 0), Gambia (34.9%; 0), Haiti (34.9%; 0), Burkina Faso (39.4%; 1), Rwanda (34.9%; 1), Chad (34.5%; 0), Cameroon (33.8%; 0), and Côte d’Ivoire (33.4%; 0). While these zinc estimates are dated, they remain the most recent consistent dataset available and continue to highlight countries where the nutritional burden is high but where country-linked publications in this field are extremely limited.

The comparison with anemia prevalence among children further reinforces the gap. Alarmingly, 62 countries report prevalence above 40%, and in 42 of them more than half of children are affected (Supplementary Material). Yet, most of these high-burden settings contribute almost no publications to the field of biofortification and micronutrient deficiencies. For instance, Mali (79.0% prevalence; 0 papers), Burkina Faso (76.6%; 1), Sierra Leone (73.4%; 1), Liberia (72.3%; 0), and Côte d’Ivoire (72.2%; 0) face some of the world’s highest anemia burdens but have virtually no country-linked studies in this research area. This mismatch underscores the absence of publications in contexts where the problem is most severe and where decision-makers urgently need country-specific guidance.

## 4. Interpreting the Gaps: Lessons for Food Security and Health Policy

This consistent pattern across iodine, zinc, and anemia highlights a simple but critical message: the countries with the heaviest nutritional burdens are often those with the least scientific evidence available to inform action.

These findings are not about statistical nuance, but about the availability of usable, local evidence [15]. Countries still lacking universal iodized-salt coverage require basic studies on retail-level testing, enforcement audits, and distribution practices [16]. Those with high zinc deficiency need analyses of the affordability and accessibility of biofortified staples, alongside research that documents barriers to adoption [17]. In contexts where anemia affects more than half of children, locally grounded evaluations of supplementation and food-based strategies are urgently needed [18]. When the country-linked publication count is limited to zero or one, this essential learning loop is broken, leaving decision-makers without the evidence required to act effectively.

The temporal trend reported in this study was included solely to contextualize the global expansion of research in this field, not to assess whether growth has been equitably distributed across regions or whether the evidence gap is narrowing over time. Evaluating geographic convergence or divergence would require a different analytical framework, including disaggregated time-series comparisons, measures of structural research capacity, and control for region-specific baseline conditions [19,20,21]. Because the purpose of this study was descriptive evidence mapping rather than modeling temporal equity, we did not analyze growth patterns by burden level or compare trends with other global health domains. Nevertheless, the sustained annual increase suggests a dynamic research landscape, and future studies could explore whether recent publication growth translates into improved geographic representation in high-burden settings [21].

This analysis has limitations. Publication counts capture only the volume of output, not the design, rigor, or quality of individual studies. Moreover, the years for iodized-salt coverage vary across countries, and zinc deficiency estimates, although the most recent available, are outdated for many contexts. These constraints were addressed by applying a consistent and transparent rule: using the latest national value available and straightforward thresholds, so that patterns could be interpreted without reliance on complex methods. While these limitations should be acknowledged, they do not alter the central conclusion: in many high-need settings, the country-linked scientific literature in this field remains scarce.

This analysis used Scopus as the sole data source because it offers broad multidisciplinary coverage across agricultural sciences, nutrition, and health, and provides detailed affiliation metadata required for territorial mapping. Comparative evaluations of literature databases have shown that Scopus indexes a wider range of journals than PubMed, particularly outside core clinical medicine, and often retrieves a higher number of documents for publication-based analyses [22,23]. Nevertheless, relying on a single database may omit studies indexed exclusively elsewhere; this potential under-coverage is acknowledged as a limitation of our study.

An important opportunity for future research is to extend this descriptive evidence-mapping approach using inferential statistical methods. Such analyses could examine how publication output relates to structural determinants, including economic indicators, research system characteristics, and regional contextual factors, and whether the observed geographic misalignments persist after accounting for these variables. Exploring these relationships through correlation analyses or regression modeling would provide deeper insight into the drivers of evidence availability and further clarify how contextual conditions shape countries’ capacity to generate or adapt research on micronutrient interventions.

**Figure 2:**
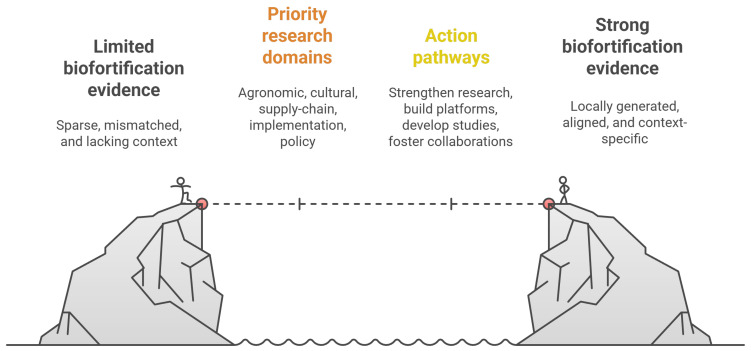
Conceptual roadmap to strengthen biofortification evidence in high-burden settings. This figure illustrates the transition from limited, mismatched, and context-insufficient biofortification evidence toward a robust, locally generated evidence base. The central section outlines priority research domains, agronomic validation, cultural and dietary assessments, supply-chain feasibility, implementation research, and policy analysis, that bridge existing gaps. Action pathways highlight strategies to operationalize this agenda, including strengthening local research capacity, developing territorially relevant studies, establishing evidence platforms, and fostering equitable international collaborations. Together, these components provide a structured roadmap to align biofortification research with nutritional burdens and support evidence-informed food security policies.

A modest shift in priorities could yield disproportionate benefits. Calls for implementation research should focus on the countries highlighted above, those with iodized-salt coverage still below 70% (and especially below 50%), or with zinc deficiency exceeding 25–35%, and with high anemia prevalence in children. Crucially, such efforts must prioritize local leadership to ensure findings are context-specific, credible, and quickly usable. Short, reproducible studies, from market testing and supply-chain audits to field-based evaluations of interventions, could be disseminated as brief reports and integrated into national monitoring systems (Figure 2) [24,25,26]. The aim is not to develop complex new indicators, but to ensure that the countries with the greatest nutritional burdens have a sufficient body of published, country-relevant evidence to support decisions with confidence [27,28,29,30,31].

From an epistemological perspective, the contribution of this study should not be interpreted in terms of statistical inference or causal estimation, but rather as a structured mapping of evidence availability and absence across territorial contexts. Descriptive evidence mapping plays a critical role in global health and food security by making visible where scientific knowledge is institutionally generated and where it remains scarce, particularly in relation to population-level needs. In this sense, the scientific value of this work lies in its capacity to identify systematic misalignments between nutritional burden and the locally linked peer-reviewed evidence base, thereby informing priorities for implementation research, capacity strengthening, and equitable knowledge production. Such analyses do not aim to replace effectiveness research, but to complement it by clarifying where context-specific evidence is most urgently required for informed policy discussion.

## 5. Conclusions

This descriptive analysis highlights a consistent geographic misalignment between global nutritional vulnerability and the territorial availability of peer-reviewed research on micronutrient deficiency mitigation strategies, including biofortification. While an absence of publications does not necessarily imply an absence of locally generated knowledge or implementation activity, the patterns observed suggest that many high-burden settings appear underrepresented in the indexed scientific literature. These findings underscore the value of strengthening research participation and visibility in regions facing the highest nutritional burdens so that the evidence base can better reflect global needs. Future work incorporating inferential analyses, study-location metadata, and broader indicators of research capacity may help clarify how structural and contextual factors shape these disparities and inform strategies to enhance geographic equity in the scientific landscape.

## Data Availability

The analyzed data will be available upon request from the corresponding author.

## Supplementary Materials

The following supporting information can be downloaded at: https://www.mdpi.com/article/10.3390/ijerph23020261/s1, Table S1: Countries and authorships, Table S2: Consumption of iodized salt, Table S3: Prevalence of zinc deficiency, Table S4: Anemia among children.

## References

[1] Passarelli S., Free C.M., Shepon A., Beal T., Batis C., Golden C.D. (2024). Global estimation of dietary micronutrient inadequacies: A modelling analysis. Lancet Glob. Health.

[2] Avnee, Sood S., Chaudhary D.R., Jhorar P., Rana R.S. (2023). Biofortification: An approach to eradicate micronutrient deficiency. Front. Nutr..

[3] Li J., Martin C., Fernie A. (2024). Biofortification’s contribution to mitigating micronutrient deficiencies. Nat. Food.

[4] Hotz C., Loechl C., de Brauw A., Eozenou P., Gilligan D., Moursi M., Munhaua B., van Jaarsveld P., Carriquiry A., Meenakshi J.V. (2012). A large-scale intervention to introduce orange sweet potato in rural Mozambique increases vitamin A intakes among children and women. Br. J. Nutr..

[5] Gupta S., Zaman M., Fatima S., Shahzad B., Brazier A.K.M., Moran V.H., Broadley M.R., Zia M.H., Bailey E.H., Wilson L. (2022). The Impact of Consuming Zinc-Biofortified Wheat Flour on Haematological Indices of Zinc and Iron Status in Adolescent Girls in Rural Pakistan: A Cluster-Randomised, Double-Blind, Controlled Effectiveness Trial. Nutrients.

[6] Ofori K.F., Antoniello S., English M.M., Aryee A.N.A. (2022). Improving nutrition through biofortification-A systematic review. Front. Nutr..

[7] Bhardwaj A.K., Chejara S., Malik K., Kumar R., Kumar A., Yadav R.K. (2022). Agronomic biofortification of food crops: An emerging opportunity for global food and nutritional security. Front. Plant Sci..

[8] Lozada-Martinez I.D., Neira-Rodado D., Martinez-Guevara D., Cruz-Soto H.S., Sanchez-Echeverry M.P., Liscano Y. (2025). Why is it important to implement meta-research in universities and institutes with medical research activities?. Front. Res. Metr. Anal..

[9] Galván-Pérez Y., Herrera-Polo M., Hernández-Páez D.A., Neira Rodado D., Salas-Navarro K., Rueda-Olivella A.M., Beltrán-Venegas T., Lozada-Martinez I.D., Delgado P. (2025). Six Sigma Applied to Healthcare: A Global Scientometrics Analysis of Health Services Quality Improvement Research. Health Serv. Insights.

[10] Lozada-Martinez I.D., Hernandez-Paz D.A., Fiorillo-Moreno O., Picón-Jaimes Y.A., Bermúdez V. (2025). Meta-Research in Biomedical Investigation: Gaps and Opportunities Based on Meta-Research Publications and Global Indicators in Health, Science, and Human Development. Publications.

[11] Our World in Data Share of Households Consuming Iodized Salt. https://ourworldindata.org/grapher/share-of-households-consuming-iodized-salt.

[12] Our World in Data Share of People Who Have Zinc Deficiency. https://ourworldindata.org/grapher/global-prevalence-of-zinc-deficiency?tab=table.

[13] Our World in Data Share of Children Who Have Anemia. https://ourworldindata.org/grapher/prevalence-of-anemia-in-children.

[14] The World Bank World Bank Country and Lending Groups. https://datahelpdesk.worldbank.org/knowledgebase/articles/906519-world-bank-country-and-lending-groups.

[15] Chu F. (2024). Implementation science: Why should we care?. J. Med. Libr. Assoc..

[16] Zimmermann M.B., Andersson M. (2021). GLOBAL ENDOCRINOLOGY: Global perspectives in endocrinology: Coverage of iodized salt programs and iodine status in 2020. Eur. J. Endocrinol..

[17] Gupta S., Brazier A.K.M., Lowe N.M. (2020). Zinc deficiency in low- and middle-income countries: Prevalence and approaches for mitigation. J. Hum. Nutr. Diet..

[18] Liu Y., Ren W., Wang S., Xiang M., Zhang S., Zhang F. (2024). Global burden of anemia and cause among children under five years 1990–2019: Findings from the global burden of disease study 2019. Front. Nutr..

[19] Cruz Rivera S., Kyte D.G., Aiyegbusi O.L., Keeley T.J., Calvert M.J. (2017). Assessing the impact of healthcare research: A systematic review of methodological frameworks. PLoS Med..

[20] Evans J.A., Shim J.M., Ioannidis J.P. (2014). Attention to local health burden and the global disparity of health research. PLoS ONE.

[21] Penfield T., Baker M.J., Scoble R., Wykes M.C. (2014). Assessment, evaluations, and definitions of research impact: A review. Res. Eval..

[22] Falagas M.E., Pitsouni E.I., Malietzis G.A., Pappas G. (2008). Comparison of PubMed, Scopus, Web of Science, and Google Scholar: Strengths and weaknesses. FASEB J..

[23] AlRyalat S.A.S., Malkawi L.W., Momani S.M. (2019). Comparing Bibliometric Analysis Using PubMed, Scopus, and Web of Science Databases. J. Vis. Exp..

[24] Rochefort G., Lapointe A., Mercier A.P., Parent G., Provencher V., Lamarche B. (2021). A Rapid Review of Territorialized Food Systems and Their Impacts on Human Health, Food Security, and the Environment. Nutrients.

[25] Lozada-Martinez I.D., Lozada-Martinez L.M., Fiorillo-Moreno O. (2024). Leiden manifesto and evidence-based research: Are the appropriate standards being used for the correct evaluation of pluralism, gaps and relevance in medical research?. J. R. Coll. Physicians Edinb..

[26] Raposo A., Saraiva A. (2025). Nutrition and Food Security for All: A Step Towards the Future. Nutrients.

[27] Xu H., Wang P., Ding K. (2024). Transforming Agriculture: Empirical Insights into How the Digital Economy Elevates Agricultural Productivity in China. Sustainability.

[28] Thompson D.K., Thepsilvisut O., Imorachorn P., Boonkaen S., Chutimanukul P., Somyong S., Mhuantong W., Ehara H. (2025). Nutrient Management Under Good Agricultural Practices for Sustainable Cassava Production in Northeastern Thailand. Resources.

[29] Armenița Arghiroiu G., Bobeică M., Beciu S., Mann S. (2025). From Crisis to Resilience: A Bibliometric Analysis of Food Security and Sustainability Amid Geopolitical Challenges. Sustainability.

[30] Abdel Aal A.M.K., Assiri M.E., Al-Farga A., Moustafa Y.M.M., Hammam A.A., Haddad S.A., Abdelkarim N.S. (2023). Exploration of the Benefits of Biofertilizers for Attaining Food Security in Egypt’s Agriculture. Agronomy.

[31] Zhao C., Geng R., Chi T., Khiewngamdee C., Liu J. (2024). Agricultural Technology Innovation and Food Security in China: An Empirical Study on Coupling Coordination and Its Influencing Factors. Agronomy.

